# Supplementary material for: Very high-speed running (VHSR) profile in elite female football: An update
Source: PLoS One. 2024 Oct 7;19(10):e0308618. doi: 10.1371/journal.pone.0308618 (PMC11458000; doi:10.1371/journal.pone.0308618)
Supplement: Attachment — Submitted filename: AnswersReviewers_PlosOne_AratzOlaizola-v2.docx [file pone.0308618.s001.docx]

June 21^st^, 2024

**Manuscript ID:** PONE-D-24-06432

**Former title:** “Very High-Speed Running (VHSR) profile in elite female football: an update”

Dear Editors of Plos One,

Thank you for your email of 9^th^ Mayr 2024 regarding our manuscript formerly entitled “Very High-Speed Running (VHSR) profile in elite female football: an update”, submitted for consideration for publication in Plos One Journal.

We resubmit a significantly revised version of our manuscript taking into account all the Reviewer’s comments. We found the Reviewer’s comments, corrections and questions very useful.

Additionally, we provide below a detailed set of point-by-point responses to the Reviewer’s comments with a precise indication of the changes introduced in the new version of the manuscript.

The main changes can be summarized as follows:

1. We have corrected and introduced all the Reviewer’s comments, corrections and questions.
2. Quality of figures was improved and new high-quality figures were added.
3. All the comments and suggestions with regard to format, ethical issues, funding and data availability have been updated according to the journal requirements.

We hope the new version of the manuscript is suitable for publication in Plos One Journal. We look forward to your response in due course.

Yours faithfully,

Aratz Olaizola

Department of Physical Activity and Sport.

Faculty of Education and Sport.

University of the Basque Country (UPV/EHU). Spain

Carretera Lasarte 71, 01007, Vitoria-Gasteiz, Spain

E-mail address: [olaizola525@gmail.com](mailto:olaizola525@gmail.com)

**Responses to Reviewer 1**

Very interesting study. I must emphasize that the order is methodologically very good. the results are clearly presented, the discussion is very detailed and interesting.

Reviewer #1: I recommend a paper prepared in this way for publication.

We thank the Reviewer for his/her valuable comments and suggestions.

**Responses to Reviewer 2**

We thank the Reviewer for his/her valuable comments and suggestions. We have corrected and introduced all your comments, corrections and suggestions and we answer below to your questions.

Next, we provide our responses to each of the comments with a detailed description of how our manuscript was modified accordingly.

Reviewer #2: This topic has excellent, valuable insights into elite female football players' very high-speed running profiles on both the theoretical and practical sides. Therefore, you selected a critical topic that is especially significant for the coaching staff of professional women's football teams: crucial for optimizing training and performance strategies in professional women's football.

You mentioned that this research has seen a considerable increase in publications. Did you explore all relevant references? The research background should be presented in more detail, adding depth to the findings by considering contextual factors.

Thank you for your comments and suggestions, we agree. We corrected it in the manuscript.

*This distribution of efforts may be influenced by contextual variables such as opponent level, match location, or score, which could affect play style [19]. The team was mainly defensive and not very dominant, with a narrow and shallow block, making coverage and short-distance defensive jumps.*

Castellano J, Blanco-Villaseñor A, Álvarez D. Contextual Variables and Time-Motion Analysis in Soccer. Int J Sports Med. 2011;32: 415–421. doi:10.1055/s-0031-1271771

Díaz-Serradilla E, Castillo D, Rodríguez-Marroyo JA, Raya González J, Villa Vicente JG, Rodríguez-Fernández A. Effect of Different Nonstarter Compensatory Strategies on Training Load in Female Soccer Players: A Pilot Study. Sports Health: A Multidisciplinary Approach. 2023;15: 835–841. doi:10.1177/19417381231176555
